# Pineal anlage tumor: clinical and diagnostic features, and rationales for treatment

**DOI:** 10.1007/s11060-023-04547-5

**Published:** 2024-01-22

**Authors:** Denise Obrecht-Sturm, Elke Pfaff, Martin Mynarek, Brigitte Bison, Martina Rodehüser, Martina Becker, Silke Kietz, Stefan M. Pfister, David T. Jones, Dominik Sturm, Andreas von Deimling, Felix Sahm, Rolf-Dieter Kortmann, Rudolf Schwarz, Torsten Pietsch, Gudrun Fleischhack, Stefan Rutkowski

**Affiliations:** 1 Pediatric Hematology and Oncology, University Medical Center Hamburg-Eppendorf, Martinistr. 52, 20246 Hamburg, Germany; 2 Hopp Children’s Cancer Center Heidelberg (KiTZ), Heidelberg, Germany; 3 Division of Pediatric Glioma Research, German Cancer Research Center (DKFZ) and German Consortium for Translational Cancer Research (DKTK), Heidelberg, Germany; 4 Mildred Scheel Cancer Career Center HaTriCS4, University Medical Center Hamburg-Eppendorf, Hamburg, Germany; 5 Diagnostic and Interventional Neuroradiology, Faculty of Medicine, University of Augsburg, Augsburg, Germany; 6 Pediatric Hematology and Oncology, Hospital Kassel, Kassel, Germany; 7 Pediatric Hematology and Oncology, Medical Department, Frankfurt am Main, Germany; 8 Department for Pediatric Hematology and Oncology, University Hospital Regensburg, Regensburg, Germany; 9 Department of Pediatric Hematology and Oncology, Heidelberg University Hospital, Heidelberg, Germany; 10 Department Neuropathology, University Hospital Heidelberg, Heidelberg, Germany; 11 Clinical Cooperation Unit Neuropathology, German Consortium for Translational Cancer Research (DKTK), German Cancer Research Center (DKFZ), Heidelberg, Germany; 12 Department for Radiation Therapy, University Medical Center Leipzig, Leipzig, Germany; 13 Department for Radiotherapy, University Medical Center Hamburg-Eppendorf, Hamburg, Germany; 14 Brain Tumor Reference Center of the German Society for Neuropathology and Neuroanatomy (DGNN), Institute of Neuropathology, University of Bonn, DZNE German Center for Neurodegenerative Diseases, Bonn, Germany; 15 Pediatrics III, University Hospital of Essen, Essen, Germany

**Keywords:** PINEAL anlage tumor, Pineoblastoma, Pineal gland

## Abstract

**Purpose:**

To provide a treatment-focused review and develop basic treatment guidelines for patients diagnosed with pineal anlage tumor (PAT).

**Methods:**

Prospectively collected data of three patients with pineal anlage tumor from Germany was combined with clinical details and treatment information from 17 published cases.

**Results:**

Overall, 20 cases of PAT were identified (3 not previously reported German cases, 17 cases from published reports). Age at diagnosis ranged from 0.3 to 35.0 (median: 3.2 ± 7.8) years. All but three cases were diagnosed before the age of three years. For three cases, metastatic disease at initial staging was described. All patients underwent tumor surgery (gross-total resection: 9, subtotal resection/biopsy: 9, extent of resection unknown: 2). 15/20 patients were alive at last follow-up. Median follow-up for 10/15 surviving patients with available follow-up and treatment data was 2.4 years (0.3–6.5). Relapse was reported for 3 patients within 0.8 years after diagnosis. Five patients died, 3 after relapse and 2 from early postoperative complications. Two-year-progression-free- and -overall survival were 65.2 ± 12.7% and 49.2 ± 18.2%, respectively. All 4 patients who received intensive chemotherapy including high-dose chemotherapy combined with radiotherapy (2 focal, 2 craniospinal [CSI]) had no recurrence. Focal radiotherapy- and CSI-free survival rates in 13 evaluable patients were 46.2% (6/13) and 61.5% (8/13), respectively.

**Conclusion:**

PAT is an aggressive disease mostly affecting young children. Therefore, adjuvant therapy using intensive chemotherapy and considering radiotherapy appears to comprise an appropriate treatment strategy. Reporting further cases is crucial to evaluate distinct treatment strategies.

**Supplementary Information:**

The online version contains supplementary material available at 10.1007/s11060-023-04547-5.

## Introduction

Pineal anlage tumor (PAT) is an extremely rare histological diagnosis first described by Schmidbauer et al. [1], not yet defined as a distinct tumor type in the most recent WHO classification of tumors of the central nervous system [2, 3]. Since the WHO classification of 2007, PAT is described as a rare variant of pineoblastoma with melanotic, cartilaginous and/or rhabdomyoblastic differentiation [4–6]. Histologically, this primary pineal tumor is characterized by heterogeneous elements of neuroepithelial and ectomesenchymal tissue, but without endodermal structures [1]. They are similar to retinal anlage tumor of the jaw, which led to its terminology [1, 7]. These tumors are thought of as highly aggressive and are associated with poor prognosis [8]. However, assumptions have been made that there are tumors which meet some characteristics of PAT, while lacking primitive features [8, 9].This led to the proposal to differentiate such tumors from the initially described tumors by Schmidbauer et al., because the latter tumors might require different adjuvant therapy strategies [8].

Overall, due to the rarity of this tumor type, reported treatment strategies are diverse and the ideal strategy e.g. using pineoblastoma protocols remains unclear [5].

Here, we report on three unpublished cases of tumors histologically classified as pineal anlage tumor and reviewed the literature focusing on clinical features and treatment.

### Patients and methods

Data of 3 patients with pineal anlage tumor treated in Germany from 2000 to 2020 were complied. This study was performed in line with the principles of the Declaration of Helsinki and from all three patients, written informed consent was obtained from patients, parents, or legal guardians for publication of their data.

DNA methylation profiling and panel sequencing was performed as part of clinical routine or relapse work-up. The Heidelberg Brain Tumor Classifier Version v12b5 (www.molecularneuropathology.com) was used for tumor classification by methylation. Next generation panel sequencing was performed analogous to procedures described by Sahm et al. [10]. Simultaneously, DNA was also derived from blood to match results with germline information.

In addition, a literature review via Pubmed.gov using “pineal anlage tumor” and “pineal” as search terms on June 9th 2022 was performed and 17 further cases of PAT were identified. For these cases, reported clinical details were analyzed in detail.

Data of the cases are summarized in Table 1. Statistical analyses were explorative and were performed using IBM^©^ SPSS® Version 25. Kaplan–Meier method was used for survival estimation.

**Table 1:**
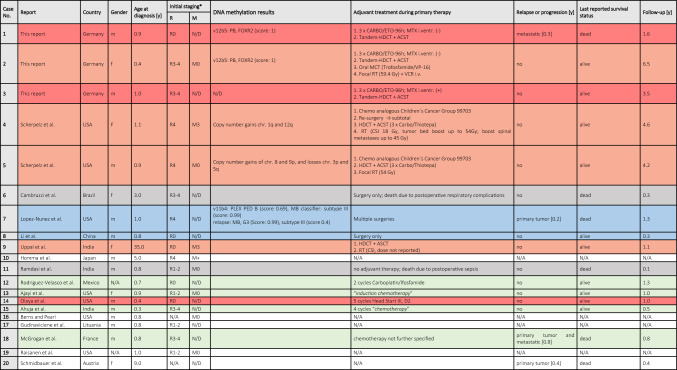
Detailed overview of all published cases of pineal anlage tumor including 3 new cases from Germany

*Initial staging was defined as status prior to start of adjuvant therapy or, if no adjuvant therapy was reported, after final surgery during initial work-up.

Colors matching the treatment strategies analogous to Figs. 2 und 3.

*f* female, *m* male, *N/A* not reported, *y* years, *R0* no residual tumor, *R1-2* near/gross total resection, *R3-4* large residual or biopsy only, *N/D* no metastases described or no report regarding metastatic status, *M0* no metastases, *i.v.* intravenously, *i.ventr.* intraventricularly, (−): not applied, ( +) applied

## Results

### Reports of 3 German cases

#### Patient 1

##### Clinical presentation

This male patient was diagnosed in 2015 at the age of 0.9 years after he presented with squinting, ataxia and signs of hydrocephalus.

##### Histology and molecular information

Histologically, the tumor was classified as PAT by the local pathologist and confirmed by national central neuropathology review. It showed the typical combination of immature neuroepithelial and ectomesenchymal components without endodermal structures and all displayed melanocytic differentiation.

**Fig. 1:**
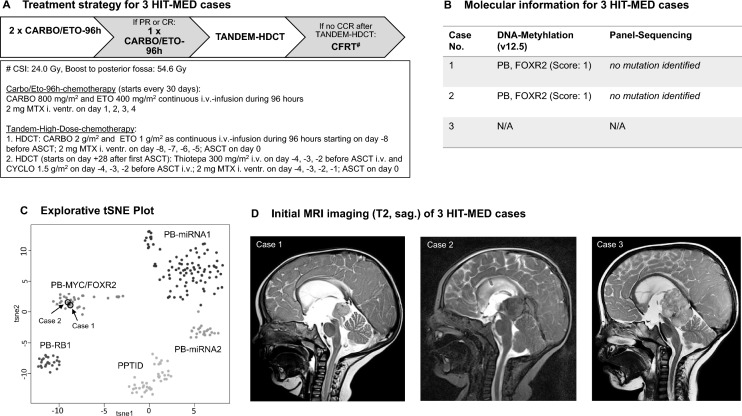
Treatment details and molecular information for HIT-MED cases. In all three cases, patients started treatment analogous to HIT-MED Guidance recommendations for pineoblastoma, which is shown here. **A** Treatment strategy for 3 HIT-MED cases, **B** Molecular information for 3 HIT-MED cases, **C** Explortive tSNE Plot, Reference cases from Liu et al., Acta Neuropathol. 2021 May; 141(5):771–785 (pineoblastoma) and Capper et al., Nature. 2018 Mar 22; 555(7697):469–474 (medulloblastoma), *PR* partial response, *(C)CR* (continuous) complete remission, *i.v.* intravenous, *i. ventr.* intraventricularly, *HDCT* high-dose chemotherapy, *ASCT* autologous stem-cell transplantation

The tumor`s methylation profile matched with PB, FOXR2 (Fig. 1B, C). Methylation profiles of cases 1 and 2 are plotted via tSNE in Fig. 1C together with published cases from molecular consensus study by Liu et al. (pineoblastoma) and Capper et al. (medulloblastoma)[14, 15]. Next generation panel sequencing did not detect any somatic mutations. Further, family history did not suggest an increased incidence of cancer in the family, therefore no genetic counselling and testing was performed.

##### Neuroradiological presentation (supplemental Fig. 1)

The initial MRI demonstrated a mass of high cellularity in the pineal region measuring an estimated volume of 5.5 ml. It was reaching into the aqueduct of Sylvius (black arrows 1.1.1–1.1.3) and causing a supratentorial hydrocephalus (1.1.1–1.1.5). A bright spot on T1WI centered in the mass (grey arrow 1.1.2) was most likely a calcification. The early postoperative MRI (1.2.1–1.1.5) displayed some postoperative change and a partly fluid, partly air-filled subdural compartment along the left cerebral hemisphere and blood clots in the resection cavity (proven by follow up). No tumor residue was detectable. An external ventricular drainage was placed in the left side ventricle (white asterisk 1.2.2). Postoperatively small bilateral hygromas along the cerebral hemispheres (wider on the right than on the left side, black asterisks), and after shunt-placement (catheter in the right side-ventricle, white asterisk) diminished width of the ventricles were documented (1.3.1–1.3.2). Next MRI (conducted shortly after treatment initiation) showed progressive disease with multiple meningeal seedings along the wall of both lateral ventricles and the third ventricle having the same signal on T2WI and restricted diffusion as the primary tumor had initially (white arrows). No local residue was detectable (1.4.1–1.4.5).

##### Treatment details, response and outcome

After complete resection, the patient received systemic chemotherapy analogous to the HIT2000 trial scheme for PNET/pineoblastoma (NCT: 00303810) [11, 12] without application of intraventricular treatment (see Fig. 1A for treatment details). The next MRI revealed new meningeal seeding (M2). As this MRI was performed early after treatment initiation, this progression was rather classified as potential progression before treatment initiation and therefore not rated as relapse for the survival analysis in this project. After 3 cycles of CARBO/ETO-96 h, tandem-high-dose chemotherapy (HDCT) with autologous stem cell transplantation (ASCT) was subsequently performed, leading to a complete remission confirmed by national central radiology review (supplemental Fig. 1, 1.5.1–1.5.5). At the end of primary treatment, the patient presented no neurological or neuro-developmental sequelae. Three months later the tumor recurred again (white arrowheads) with cranial (white arrows) and spinal metastases (Black arrows; supplemental Fig. 1, 1.6.1–1.6.5). Relapse therapy was performed using metronomic treatment *RIST* (sirolimus, irinotecan, dasatinib, temozolomide) [13]. This increased rapidly on follow up (supplemental Fig. 1, 1.7.1–1.7.5) partly with laminar (white arrowhead) and nodular lesions (white small arrows, supplemental Fig. 1, 1.7.2 and 1.7.5), partly with broad bands of confluent dissemination demonstrating an intense restriction of diffusion (white arrows, supplemental Fig. 1, 1.7.1, 1.7.3, 1.7.4) like the primary tumor but only little contrast enhancement as known from other aggressive tumors after therapy (e.g. in medulloblastomas). Despite the low quality of the spinal MRI the progression becomes obvious (white arrows supplemental Fig. 1, 1.7.5). Radiotherapy (RT) was discussed, but due to the extent of metastases and age considered as contraindicated. He died from disease progression one year after the end of first-line therapy.

#### Patient 2

##### Clinical presentation

This 0.4-year-old girl was diagnosed with pineal anlage tumor in 2016. She presented with signs of hydrocephalus, dehydration after massive vomiting, myoclonies and sunset phenomenon.

##### Histology and molecular information

Histological presentation of the tumor matched to the tumor from patient 1 and was again confirmed by central review. The tumor’s methylation profile also matched with PB, FOXR2 (Fig. 1B, C). Next generation panel sequencing did not detect any somatic mutations and family history did not suggest an increased incidence of cancer in the family, therefore no genetic counselling and testing was performed.

##### Neuroradiological presentation (supplemental Fig. 2)

**Fig. 2:**
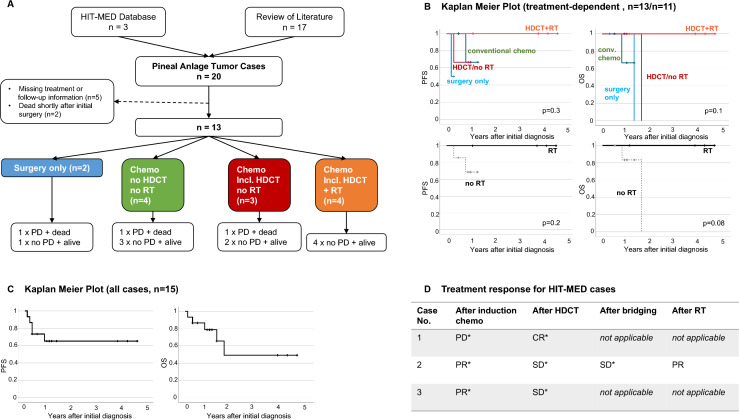
Clinical courses and Kaplan Meier Survival Estimation. A Overview of individual cases` clinical course incl. treatment and outcomes, B Kaplan–Meier Plot (treatment-dependent) for n = 13 cases (above) with available treatment and follow-up data (n = 2 excluded due to death from early postoperative complications) and for n = 11 cases (below) with adjuvant treatment (n = 2 excluded due to surgery only), C Kaplan–Meier Plot (all cases with available treatment and follow-up data), D Treatment response for HIT-MED cases. *centrally reviewed. *PD* progressive disease, *SD* stable disease, *PR* partial response, *CR* complete remission, *HDCT* high-dose chemotherapy, *RT* radiotherapy

This patient presented with a tumor of the pineal region. It showed intermediate to mildly elevated signal on T2 weighted images (T2WI) and bleeding interfering with the diffusion weighted imaging. It demonstrated an inhomogeneous intermediate contrast enhancement. The aspects resemble other tumors of high cellularity in this localization such as pineoblastoma. In contrast to most germ cell tumors in this localization, perifocal edema was not observed. Initially, the tumor was only biopsied. Before adjuvant treatment initiation, rapid progression occurred and second surgery with the aim of maximal save resection was performed; residual tumor with an estimated volume of 23.9 ml remained. Spinal MRI and CSF by lumbar puncture showed no evidence for metastases.

##### Treatment details, response and outcome

The patient received 3 cycles of CARBO/ETO-96 h without application of intraventricular treatment, leading to a partial response (PR). Regarding the residual tumor mass, biopsy was performed showing no vital tumor cells. Tandem-high-dose chemotherapy with autologous stem cell transplantation was performed, resulting in a stable disease. The second HDCT course (thiotepa and cyclophosphamide i.v., methotrexate (MTX) intraventricularly) was complicated by hepatic veno-occlusive disease, which was successfully treated without long-term complications. Afterwards, the patient received oral maintenance therapy (6 cycles trofosfamide / etoposide orally and etoposide intraventricularly) until she reached the age of 18 months and qualified for focal RT. During this time, the tumor was stable. Focal RT was performed using proton beam up to 59.4 Gy to the residual tumor volume. Vincristine was administered intravenously simultaneously on a weekly basis. She was alive at last follow-up 6.5 years after initial diagnosis without further therapy. Residual MRI findings are interpreted as scar tissue. At this time, she did not have any residual neurological or neuropsychological residual. Notably, the girl was found to have a secondary malignancy 6 years after diagnosis of PAT. Follow-up MRI showed a lesion in the right cerebellar hemisphere (supplemental Fig. 2.5). Biopsy was performed and revealed a high grade glioma. Thereafter, she was lost to follow-up.

#### Patient 3

##### Clinical presentation

The pineal anlage tumor was diagnosed for this male infant in 2018 at the age of 1.0 years. Symptoms were signs of hydrocephalus occlusus, Parinaud`s syndrome and fatigue.

##### Histology and molecular information

Histologically, the tumor was also classified as PAT by the local pathologist and confirmed by national central neuropathology review and showed the typical combination of immature neuroepithelial and ectomesenchymal components without endodermal structures and all displayed melanocytic differentiation, but molecular results were implausible. Unfortunately, even re-assessment of a second tumor sample and matching blood were analyzed resulting in implausible results again. We therefore assumed that the tumor samples sent for molecular diagnostic did not belong to patients 3. Further tumor material was not available.

Next generation panel sequencing didn’t reveal an evidence for germline mutations in the genes covered by this panel. Further, family history did not suggest an increased incidence of cancer, therefore no genetic counselling and testing was performed.

##### Neuroradiological presentation (supplemental Fig. 3)

**Fig. 3:**
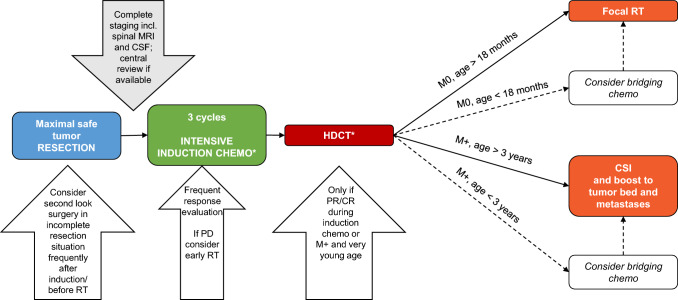
Proposed flowshart to help with future therapeutic decision making for Pineal Anlage Tumor. *according to national standards. *CSF* cerebrospinal fluid, *RT* radiotherapy, *CSI* craniospinal irradiation, *HDCT* high-dose chemotherapy, *PD* progressive disease, *PR* partial response, *CR* complete remission, M0: localized disease/no metastases, M + : disseminated disease

The patient presented with a large tumor reaching from the pineal region supratentorielly into the posterior part of the third ventricle and infratentorielly into the tectal region and the quadrigeminal cistern, compressing the brainstem and cerebellum (Fig. 1D) with radiologically unclear origin. As in patients 1 and 2 the tumor again showed intermediate to mildly elevated signal on T2 weighted images (T2WI), and intermediate to restricted diffusion as a sign of high cellularity as well as inhomogeneous intermediate contrast enhancement. Partial resection was performed with three small residues remaining. Cranial and spinal MRI did not show metastases by national central imaging review.

##### Treatment details, response and outcome

The patient received 3 cycles of CARBO/ETO-96 h systemic chemotherapy and intraventricular MTX (2.0 mg absolute single dose, repetitive every 24 h on day 1–4) and achieved a partial response. It was followed by tandem-high-dose chemotherapy and autologous stem cell transplantation. MRI response monitoring showed stable disease, so a national neurosurgical review panel was summoned. The panel recommended re-surgery only in case of progressive disease. 3.1 years after initial surgery the patient is still alive and without relapse. He still suffers from Parinaud`s syndrome and associated coordination difficulties. Yet, his intellectual development appears age-appropriate.

### Demographic details of the assembled cohort

Taking into account all cases of PAT identified in the literature and the three additional German cases, in 2023, there are 20 known and reported cases (Table 1) [1, 5, 8, 9, 16–27]. Cases were published from all over the world starting in 1989 until July 2022. Age at diagnosis ranged from 0.3 to 35.0 years. All but three cases were diagnosed before the age of 3 years. All but one case affected pediatric patients. The tumor affected predominantly male patients with a male/female ratio of 2:1.

### Initial staging and surgery

In all cases, tumors originated in the pineal region, affecting infra- and supratentorial structures. Dissemination at initial diagnosis was reported for three cases and showed spinal involvement in two. In all cases, surgery was the first therapeutic element leading to the histological diagnosis of pineal anlage tumor. Three patients underwent re-surgery prior to start of adjuvant treatment. Further, in two patients, re-surgery was performed during adjuvant chemotherapy and another patient was treated with multiple surgeries only. Overall, complete resection was reported for five patients and gross-total resection for a total of 9 patients.

#### Postoperative clinical courses

Information about postoperative adjuvant treatment was available for 15/20 (75%) cases. Two patients died shortly after surgery due to postoperative complications (sepsis, respiratory complication). For another two patients, available information indicated that no adjuvant chemo- or radiotherapy was applied. One of them received multiple surgeries. Thus, the subsequent analysis was performed on 11 cases who had received adjuvant treatment. In all 11 cases, treatment regimens consisted of intensive chemotherapy of different national standards (Table 1). All three patients from Germany, on which we have reported here, received the same induction chemotherapy with CARBO/ETO-96 h followed by tandem-HDCT and ASCT. Treatment response for these patients is displayed in Fig. 2D. Further, 4 more patients from the literature received HDCT with ASCT after induction chemotherapy resulting in a series of 7 cases. In one patient hepatic sinusoidal obstruction syndrome occurred.

Four patients received radiotherapy, all after HDCT. RT was focal to the tumor bed for two non-metastasized cases and consisted of a craniospinal irradiation (CSI) followed by boosts to the tumor bed and metastases for two patients with spinal dissemination. Irradiation dosage is displayed by Table 1, if reported.

Oral maintenance chemotherapy was used as bridging for one patient between HDCT and focal RT until the age of 18 months.

Outcome and postoperative clinical courses were reported for 15/20 cases (missing information for cases 10, 16, 17, 19 and 20). For these cases, 2-year progression-free survival (PFS) was 65.2 ± 12.7% (Fig. 2C). Estimated 2-year overall survival (OS) was 49.2 ± 18.2%.

#### Treatment-related outcomes

Two additional cases who died from direct postoperative complications (cases number 6 and 11) were excluded from the further treatment-focused survival analyses, leaving 13 patients in the study cohort. Relapse or disease progression occurred in 3/13 patients. Median time to relapse or progression was 0.4 (0.2–0.8) years. All three patients with relapse or disease progression died. Median follow-up for 10 surviving patients was 2.4 years (0.3–6.5). All 4 patients who received high-dose chemotherapy combined with RT (focal RT: 2, CSI: 2) had no recurrence and were alive at last follow-up (Fig. 2B). Nevertheless, there were also patients, who survived their disease after surgery only (n = 1), conventional chemotherapy combined with RT (n = 3) or chemotherapy including HDCT without RT (n = 3). Patients who experienced relapse and died subsequently due to disease progression where treated with surgery only (n = 1), additional chemotherapy without HDCT (n = 1) and HDCT without RT (n = 1).

Focal RT- and CSI-free survival rates were 46.2% (6/13) and 61.5% (8/13), respectively.

Kaplan Meier survival estimation showed a trend for superior survival for patients, who received RT (focal or CSI) during their primary treatment (p = 0.08; Fig. 2B).

## Discussion

This project aimed at evaluating treatment strategies for PAT. As these tumors histologically can be clearly distinct from other pineal region tumors, the question is, whether these tumors should be treated similar to other pineal region tumors e.g. pineoblastoma. As specific risk-adapted treatment strategies for specific molecular pineoblastoma subgroups may be established in the future, it appears reasonable to independently analyze treatment strategies for PAT.

To date, most case reports focused on histological descriptions with frequently missing clinical details and short follow-up times. Furthermore, publication bias cannot be excluded. These conditions only allow for very limited statistical analyses and conclusions for treatment recommendations need to be drawn with caution.

Nevertheless, sharing information about the clinical presentation and disease course of pineal anlage tumor is important. Therefore, we summarized all published cases and their clinical information.

Pineal anlage tumor primarily mostly affects young children during the first years of life. Analogous to other childhood embryonal brain tumor entities as e.g. medulloblastoma, males are affected more often than females.

A detailed staging process including spinal MRI and CSF cytology should always be part of the initial clinical work-up of these patients as already suggested by Ajayi et al. [5].

Radiologically, PATs are often of large size at first diagnosis and present with circulatory dysfunction of the cerebrospinal fluid and consecutive occlusive hydrocephalus. Furthermore, these tumors show heterogeneous enhancement. Using computer tomography, the tumor is hyperdense, often cystic or calcified. In MRI, the tumor is T1-iso- or hypointense and T2-isointense compared to the surrounding brain parenchyma, and shows diffusion restriction [5, 8, 24].

Nevertheless to date, no specific radiological characteristics are described to identify PAT by MRI only [5]. Therefore, upfront surgery with the aim to obtain a tissue-based diagnosis seems to be reasonable in pineal masses negative for germ-cell tumor markers AFP and beta-HCG as well as negative medical history of a trilateral retinoblastoma.

In the reported German cases, biological relationship to pineoblastoma (PIN MYC/FOXR2) was found. Apart from this, similarity regarding the methylation signature of choroid plexus tumor at initial diagnosis and medulloblastoma at relapse for one case was described by Lopez-Nunez et al. [18] (case 7 of this series). In fact, further biological characterized cases need to be reported to draw a solid conclusion regarding the biology of pineal anlage tumor.

As pineal region tumors are challenging to resect, addressing a specialized neurosurgical center needs to be considered whenever possible. Maximal save resection should be strived for, since PAT seem to show a wide range of response to adjuvant therapy and small tissue samples might show a non-representative part of the tumor resulting in a false diagnosis [5, 23]. Taking into account the high chance of recurrence, from the author`s point of view, adjuvant treatment is indicated for all affected patients: PFS and OS are not yet satisfying based on this small series.

Since radiotherapy—and especially CSI that is usually considered necessary to treat embryonal tumors of the CNS – is associated with neurocognitive sequelae, when used in very young patients, intensive intravenous chemotherapy appears to be a reasonable choice. In reported cases, treatment protocols for embryonal tumors (formerly “primitive neuroectodermal tumors”) or pineoblastoma were chosen.

In this series, two patients with non-metastatic PAT and good response to induction chemotherapy were treated with focal radiotherapy after HDCT and did not relapse within the observation period. Therefore, we believe that focal RT might be considered as an additional treatment modality in patients with localized disease even at young age > 18 months. However, present data indicate that pineal anlage tumor can spread along the CNS especially during relapse. Therefore, craniospinal irradiation seems reasonable for disseminated disease, when it is not contraindicated due to young age or other conditions.

On the basis of the reported experience, we developed a flowchart to help making therapeutic decisions in these rare cases (Fig. 3). However, the optimal dose prescription and schedule within the multimodal treatment concept is still unclear and should be oriented to the recommendation and experiences of other embryonal CNS tumors in young children. Further, against the background of potential biological relationship to pineoblastoma and the fact that established treatment strategies for pineoblastoma / embryonal brain tumors appear to be efficient also in PAT, one may also discuss to follow national and international guidelines for pineoblastoma. Still, as specific treatment strategies may emerge for specific molecular subgroups of pineal region tumors, it appears reasonable to consider PAT also independently.

Finally, more knowledge needs to be obtained regarding the clinical presentation, molecular characteristics and treatment strategies of pineal anlage tumor to develop solid treatment guidelines. Therefore, we would like to encourage clinicians to share their experiences with patients suffering from rare tumors like pineal anlage tumor.

### Supplementary Information

Below is the link to the electronic supplementary material.

Supplementary file1 (PDF 1825 KB)

## Data Availability

The datasets generated during and/or analysed during the current study are available from the corresponding author on reasonable request.
