# Supplementary material for: Pineal anlage tumor: clinical and diagnostic features, and rationales for treatment
Source: J Neurooncol. 2024 Jan 22;166(2):359–68. doi: 10.1007/s11060-023-04547-5 (PMC10834633; doi:10.1007/s11060-023-04547-5)
Supplement: Supplementary file 1 — Supplementary file1 (PDF 1825 KB) [file 11060_2023_4547_MOESM1_ESM.pdf]

Supplemental Figure 1: Representative MRI scans of patient 1

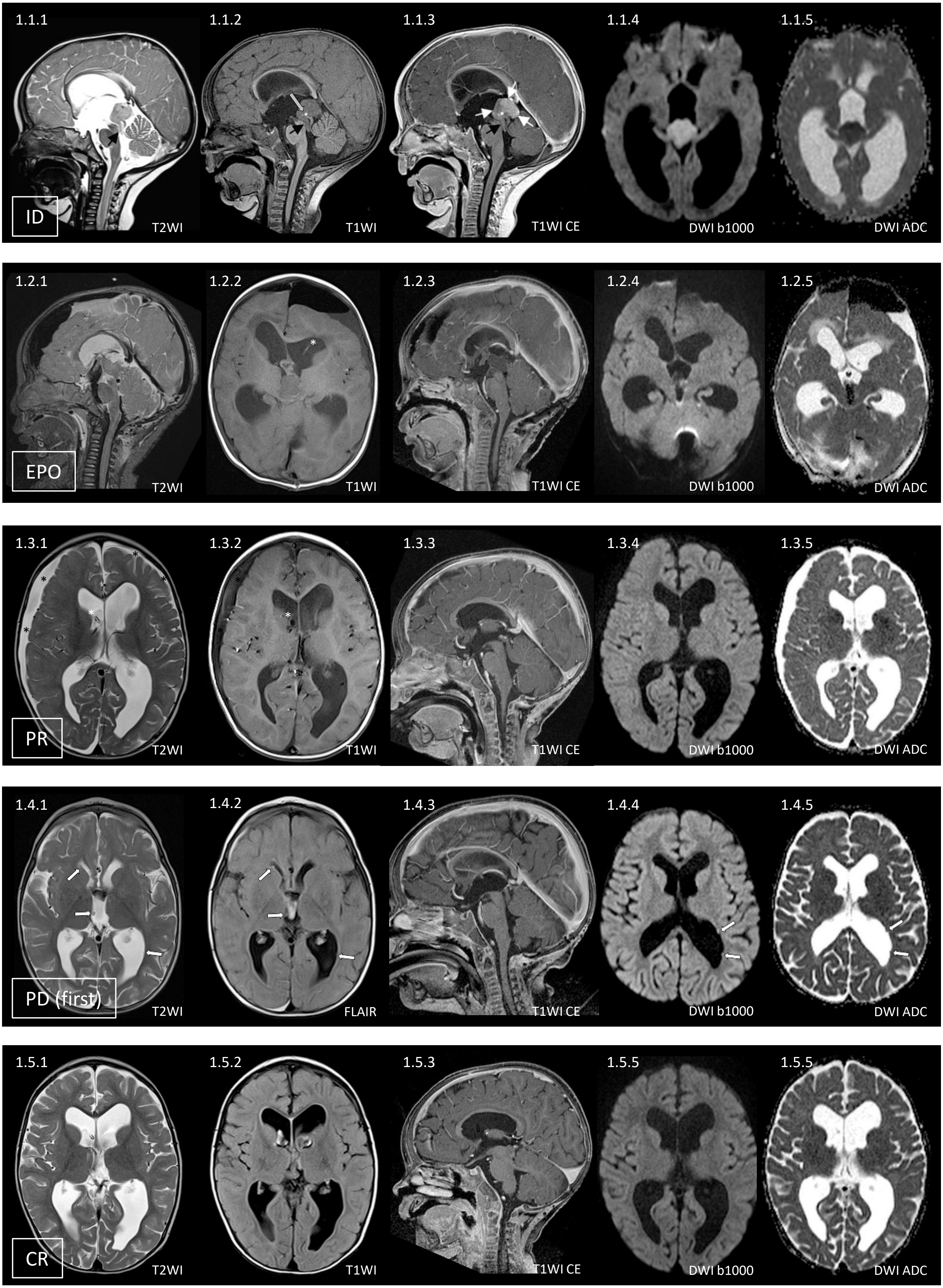

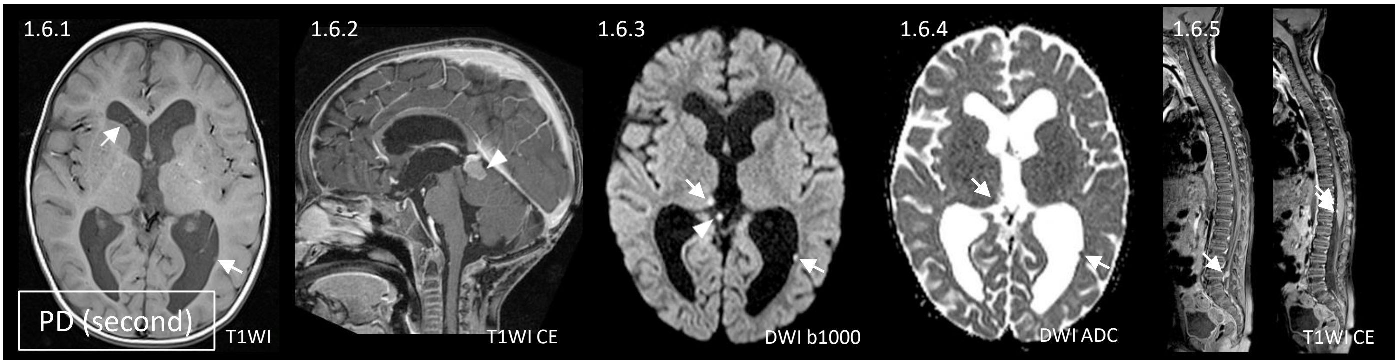
1.6.1
PD (second)
T1WI
1.6.2
T1WI CE
1.6.3
DWI b1000
1.6.4
DWI ADC
1.6.5
T1WI CE

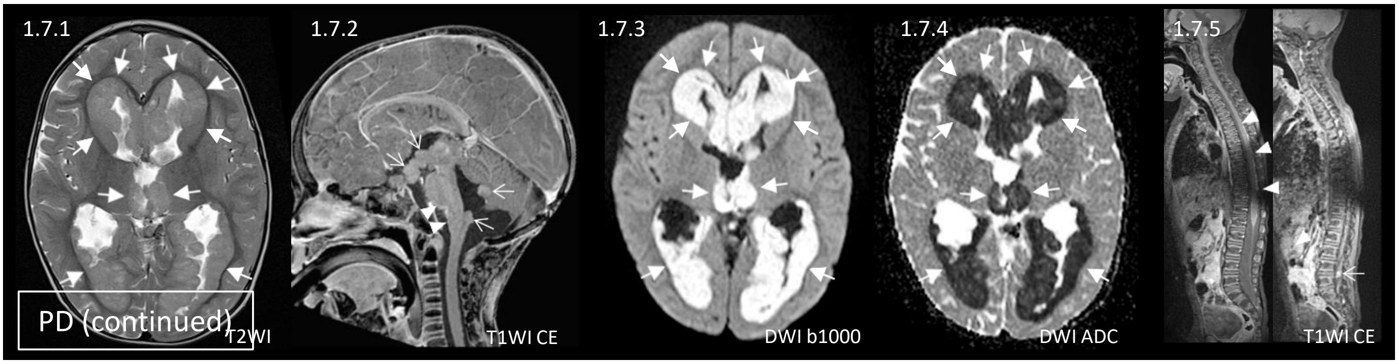
1.7.1
PD (continued)
T2WI
1.7.2
T1WI CE
1.7.3
DWI b1000
1.7.4
DWI ADC
1.7.5
T1WI CE

Supplemental Figure 2: Representative MRI scans of patient 2

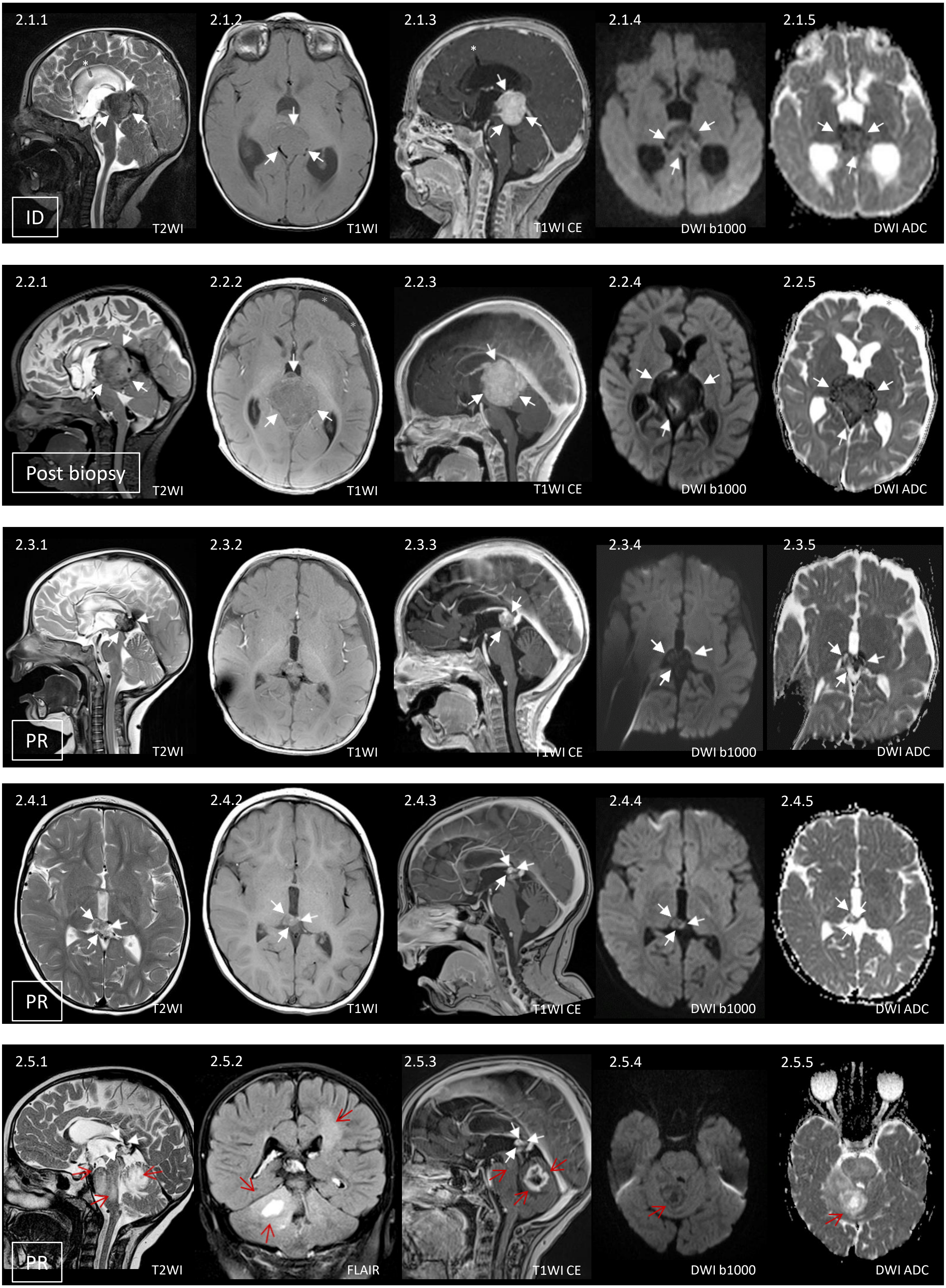

Supplemental Figure 3: Representative MRI scans of patient 3

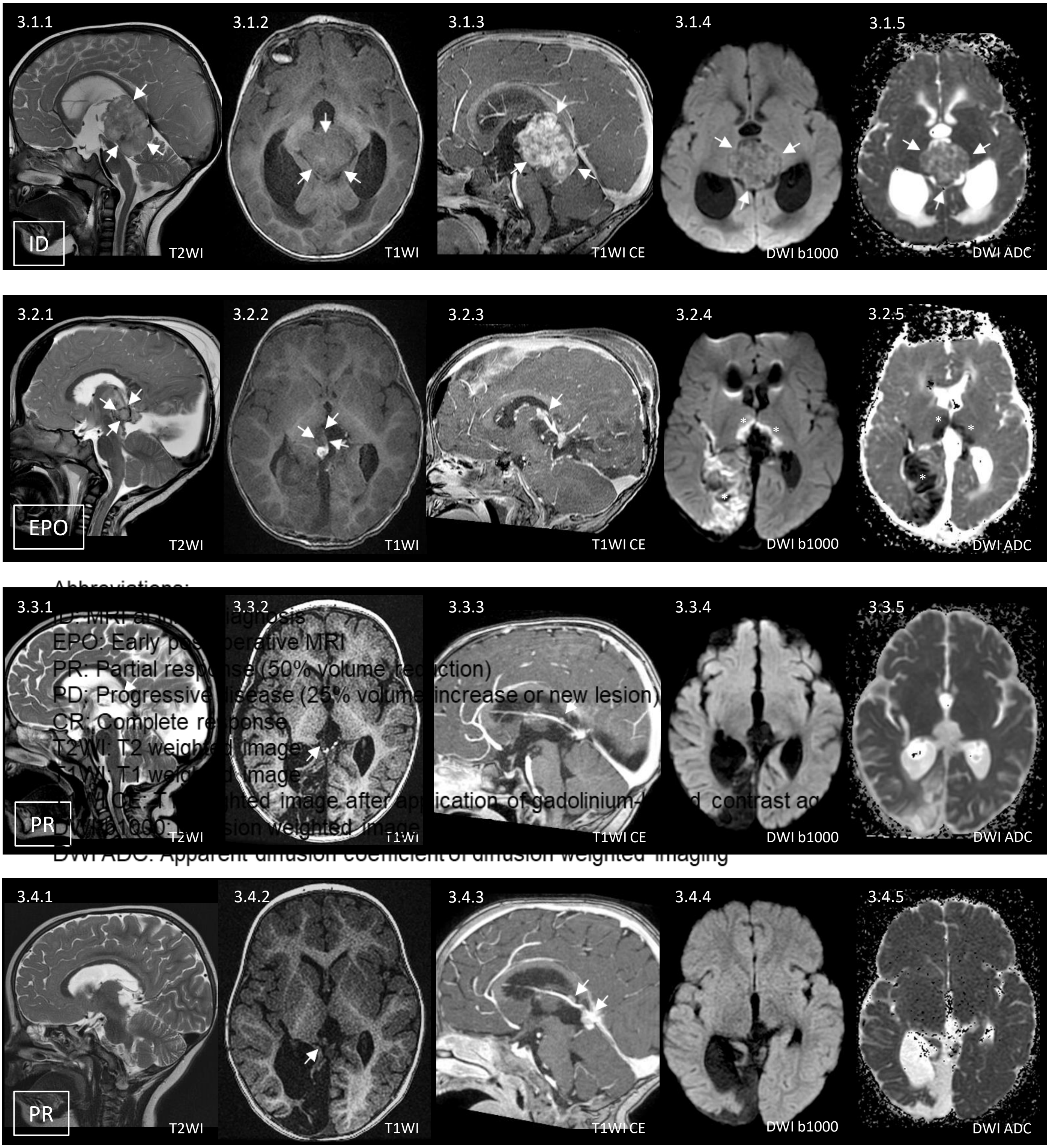

Abbreviations:
ID: MRI at initial diagnosis
EPO: Early postoperative MRI
PR: Partial response (50% volume reduction)
PD: Progressive disease (25% volume increase or new lesion)
CR: Complete response
T2WI: T2 weighted image
T1WI: T1 weighted image
T1WI CE: T1 weighted image after application of gadolinium-based contrast agent
DWI-b1000: Diffusion weighted image b 1000
DWI ADC: Apparent diffusion coefficient of diffusion weighted imaging

Patient 1 (supplemental figure 1)

The initial MRI demonstrated a mass of high cellularity in the pineal region measuring an estimated volume of 5.5 ml. It was reaching into the aqueduct of Sylvius (black arrows 1.1.1-1.1.3) and causing a supratentorial hydrocephalus (1.1.1-1.1.5). A bright spot on T1WI centered in the mass (grey arrow 1.1.2) was most likely a calcification. The early postoperative MRI (1.2.1-1.1.5) displayed some postoperative change and a partly fluid, partly air-filled subdural compartment along the left cerebral hemisphere and blood clots in the resection cavity (proven by follow up). No tumor residue was detectable. An external ventricular drainage was placed in the left side ventricle (white asterisk 1.2.2). Postoperatively small bilateral hygromas along the cerebral hemispheres (wider on the right than on the left side, black asterisks), and after shunt-placement (catheter in the right side-ventricle, white asterisk) diminished width of the ventricles were documented (1.3.1-1.3.2). Next MRI showed progressive disease with multiple meningeal seedings along the wall of both lateral ventricles and the third ventricle having the same signal on T2WI and restricted diffusion as the primary tumor had initially (white arrows). No local recurrence was detectable (1.4.1.-1.4.5). Therapy was continued leading to complete remission (CR) (1.5.1-1.5.5). Less than three months later, an early local recurrence (white arrowheads) with meningeal dissemination (white arrows) in cranial and spinal localization (black arrows) occurred (1.6.1-1.6.5). This increased rapidly on follow up (1.7.1-1.7.5) partly with laminar (white arrowhead) and nodular lesions (white small arrows, 1.7.2 and 1.7.5), partly with broad bands of confluent dissemination demonstrating an intense restriction of diffusion (white arrows 1.7.1, 1.7.3, 1.7.4) like the primary tumor but only little contrast enhancement as known from other aggressive tumors after therapy (e.g. in medulloblastomas). Despite the low quality of the spinal MRI the progression becomes obvious (white arrows 1.7.5).

MRI scans of patient 2 (supplemental figure 2)

The first MRI available (2.1.1-2.1.5) showed a pineal tumor with acute hemorrhage, measuring about 10.56 ml (white arrows). An external ventricular drainage was placed (asterix). Thirteen days later, the tumor size had increased to 23.9 ml (2.2.2-2.2.5). After biopsy, hygroma was visible along the left hemisphere (grey asterixis 2.2.2 and 2.2.5) and diminished successively during therapy (2.3.1-2.3.5). After more than 5 years of follow up, there was only a stable small contrast enhancing residue without definite restricted measuring about 0.5 ml (2.4.1-2.4.5). As there were still blood residues, the diffusion weighted imaging is not helpful to differentiate between vital tumor and scar, due to its stable size it is rated as a scar.

Five years after the initial diagnosis (2.5.1-2.5.5) the girl developed a cerebellar and hemispheric manifestation as second malignancy (red arrows). The pineal residue was still the same (white arrows).

MRI scans of patient 3 (supplemental figure 3)

Initially, the MRI demonstrated a tumor in the pineal region of 34.4 ml, reaching anteriorly into the third ventricle and posteriorly into the quadrigeminal cistern compressing mesencephalon and upper part of the cerebellum. The tumor was inhomogeneous with intermediate signal on T2WI and contrast enhancement and displayed an inhomogeneous intermediate diffusion (3.1.1-3.1.5). After subtotal removal, severe postoperative change was visible, with blood-clots in the resection cavity and infarction of the territory of the right posterior cerebral artery, including parts of the thalami. Tumor were left in two localizations, one along the fornix, the other in the pineal region between the internal veins (3.2.1-3.2.5). On follow up, the infarct underwent partly cystic and partly necrotic change (3.3.1-3.3.5) and the two small tumor residues reached PR under chemotherapy the lesion along the fornix 3.3.1) just as the residue in the pineal region between the internal veins (3.3.3). Further shrinkage was documented, and the residues were voted as scar on long follow up (3.4.1-3.4.5).
